# Disruption of FGF Signaling Ameliorates Inflammatory Response in Hepatic Stellate Cells

**DOI:** 10.3389/fcell.2020.00601

**Published:** 2020-07-22

**Authors:** Cong Wang, Yuelong Li, Hao Li, Yali Zhang, Zhangguo Ying, Xuye Wang, Tingting Zhang, Wenshu Zhang, Zhichao Fan, Xiaokun Li, Jisheng Ma, Xuebo Pan

**Affiliations:** School of Pharmaceutical Sciences, Wenzhou Medical University, Wenzhou, China

**Keywords:** FGFR, MMP9, hepatic stellate cell, inflammation, NF-κB

## Abstract

It is a well-documented event that fibroblast growth factors (FGFs) regulate liver development and homeostasis in autocrine, paracrine, and endocrine manners via binding and activating FGF receptors (FGFRs) tyrosine kinase in hepatocytes. Recent research reveals that hepatic stellate cells (HSCs) play a fundamental role in liver immunology. However, how FGF signaling in HSCs regulates liver inflammation remains unclear. Here, we report that FGF promoted NF-κB signaling, an inflammatory pathway, in human HSCs, which was associated with FGFR1 expression. Both FGF and NF-κB signaling in HSCs were compromised by FGFR1 tyrosine kinase inhibitor. After stimulating HSCs with proinflammatory cytokines, expression of multiple FGF ligands was significantly increased. However, disruption of FGF signaling with FGFR inhibitors prominently reduced the apoptosis, inflammatory response, NF-κB nuclear translocation, and expression of matrix metalloproteinase-9 (MMP-9) induced by TNFα in HSCs. Interestingly, FGF21 significantly alleviated the inflammation responses in the concanavalin A (Con A)-induced acutely injured liver. Unlike canonic FGFs that elicit signals through activating the FGFR–heparan sulfate complex, FGF21 activates the FGFR–KLB complex and elicits a different set of signals. Therefore, the finding here indicates the urgency of developing pathway-specific inhibitors that only suppress canonical FGF, but not non-canonical FGF21, signaling for alleviating inflammation in the liver, which is presented in all stages of diseased liver.

## Introduction

Inflammatory reactions drive multiple cellular processes in the chronically diseased liver, particularly in liver fibrosis that is a highly conserved response to hepatic injury (Pradhan-Sundd et al., 2018). It occurs in patients with many kinds of liver diseases, such as chronic viral hepatitis, non-alcoholic fatty liver disease (NAFLD), alcoholic liver disease (ALD), and cholestatic and autoimmune liver disease. The distinct characteristic feature in the process of liver fibrosis is the transformation of hepatic stellate cells (HSCs) from quiescent and lipid-storing cells to activated and extracellular matrix (ECM)-producing cells (Lei et al., 2016). The links between hepatic inflammation and fibrosis have been intensively studied. Yet, the underlying mechanism is still not well documented.

Quiescent HSCs are located in the Disse space near the sinusoid endothelial cells (SEC) and hepatocytes. They are vitamin A-storing cells that can transdifferentiate to ECM-producing cells in response to stimuli released during inflammation (Tao et al., 2019). The continuous activation of HSCs by cytokines released from damaged hepatocytes and microenvironment plays a vital part in the development of hepatic fibrosis (Salloum et al., 2016). Treating murine or human HSCs with proinflammatory cytokines and lipopolysaccharide (LPS) dramatically activates the nuclear factor kappa B (NF-κB) signal pathway and subsequently the production of chemokines and cytokines (de Mingo et al., 2016; Bruschi et al., 2017). In response to liver injury, HSCs undergoes transdifferentiated into myofibroblastic cells expressing α-smooth muscle actin (αSMA), and subsequently a large amount of collagen and proinflammatory cytokines, including TNFα, IL-6, MCP-1, and TGFβ (Dewidar et al., 2019). In addition, many kinds of matrix metalloproteinases (MMPs) are also expressed in HSCs in acute hepatic damage, which contributes to cell migration and differentiation during wound healing process (Roderfeld, 2018).

Fibroblast growth factor (FGF) is one of the major growth factor families that increase expression in liver injury. The FGF family consists of 18 receptor-binding ligands that control a broad spectrum of cellular processes. FGFs exert their regulatory signals by activating FGF receptor (FGFR) tyrosine kinases, which are encoded by four highly homologous genes (Faham et al., 1998; Klint and Claesson-Welsh, 1999; Ornitz, 2000; Powers et al., 2000; McKeehan et al., 2009; Li et al., 2016). Upon activation, FGFRs elicit both canonical and non-canonical signals. The canonical signals include FRS2α-dependent activation of ERK and PI3K, as well as FRS2α-independent activation of PLC-γ (Zhang J. et al., 2008; Zhang Y. et al., 2008; Zhang et al., 2010, 2012a,b; Lin et al., 2011; Li et al., 2016; Wang and Wang, 2016; Wang et al., 2019); non-canonical pathways, which are poorly characterized, include post-translational modification of LDHA and TAK1, which increases the stability and activity of these molecules (Zhang et al., 2004; Lan et al., 2008; Fan et al., 2011; Jin et al., 2017; Liu et al., 2018; Wang et al., 2018). Our recent data showed that non-canonical signaling likely accounts for the receptor isoform-specific signals.

Both FGF and FGFR are expressed in a highly spatiotemporal and cell type-specific pattern. Ectopic expression of FGF and FGFR isoforms has been identified as culprits for multiple diseases (Li et al., 2016; Wang et al., 2019). *In vitro* experiments show that transdifferentiated HSCs secreted FGF2, FGF7, and FGF9 (Itoh et al., 2016). Moreover, expression of FGF1 in HSCs has been implicated in diverse biological processes, including hepatic development and regeneration (Peterova et al., 2016). The IIIc splice variant of FGFR isoforms is expressed in freshly isolated primary rat HSCs (Mohammadalipour et al., 2019). It is noteworthy that FGF2 secreted from HSCs and hepatocytes markedly activates FGFR signaling in both autocrine and paracrine manners at the damaged liver (Zhang et al., 2017). Besides accelerating regeneration in the injured liver, FGF signaling also leads to fibrosis development. There is evidence that as a commonly used model of chronic liver injury, carbon tetrachloride (CCl4)-induced liver fibrosis is significantly reduced in FGF1/FGF2-deficient mice (Kou et al., 2019). Consistently, inhibition of FGF signaling blunts inflammatory through restraining activation of the NF-κB signaling cascades in several experimental models for chronic inflammatory diseases (Huang et al., 2019; Qi and Xin, 2019).

In the present study, we aimed to assess the potential of FGFR to serve as a novel target for controlling inflammation in the injured liver and HSC transdifferentiation. We herein showed that FGF signal was induced by TNFα in human stellate cells. Suppression of FGFR signaling restrained adhesion of monocyte to HSCs through inhibiting secretion of proinflammatory cytokines and MMPs expression, especially MMP9 activation. We also demonstrated that FGF21 was dramatically upregulated after FGFR inhibition, which limited the inflammation and suggested a negative feedback control by FGF signaling. The study reveals that inhibition of FGF signal at the inflammatory stage alleviates fibrosis progression through inhibiting HSC activation.

## Materials and Methods

### Animals

The mice were housed in a pathogen-free facility at the Wenzhou Medical University, with an ambient temperature of 23 ± 3°C, relative humidity of 55 ± 10%, and 12-h light/12-h dark cycle. Mouse procedures were approved by the Program of Animal Resources of the Wenzhou Medical University in accordance with the principles and procedure of the *Guide for the Care and Use of Laboratory Animals*. Transgenic FGF21 mice were bred and genotyped as previously described (Trokovic et al., 2003; Yu et al., 2003; Lin et al., 2007a, b; Lee et al., 2014). The 10- to 12-week-old FGF21 transgenic mice and wild-type littermates were used in the experiments. For liver inflammation model, 6.5 mg/kg concanavalin A (Con A) saline was administered via tail vein injection. The mice were sacrificed 8 h after the injection. As controls, mice were injected with saline alone (Tiegs et al., 1992).

### Cell Culture System

The LX-2 cells (from ATCC) were tested for mycoplasma contamination. The negative cells were cultured in DMEM medium with 10% FBS, 100 U/ml penicillin, and 100 mg/ml streptomycin at 37°C in a 5% CO_2_ humidified incubator. The cells were plated in six-well tissue culture dishes cultured until 70–80% confluency. The cells were then subjected to overnight starvation in serum-free DMEM, washed with PBS to remove non-attached cells and cell debris, and treated with 10 ng/ml TNFα and/or 100 nM AZD4547 for the required time. The control groups were treated with solvents only.

### Western Blotting Analysis

Cells were lysed with the RIPA buffer (25 mM Tris–HCl, 150 mM NaCl, 1% Non-idea P-40, 1% sodium deoxycholate, and 0.1% sodium dodecyl sulfate) with PMSF and phosphatase inhibitors. After centrifugation at 12,000 *g* and 4°C for 15 min, the supernatants were collected. After determination of the total protein concentration, equal amount of samples was separated by 10% SDS-PAGE gel electrophoresis and electro-transferred to a 0.45-μm polyvinylidene difluoride membrane. The membranes were blocked in TBST containing 5% non-fat milk for 1.5 h at room temperature and incubated with the following antibodies overnight at 4°C. The source and dilution of each antibody are as follows: anti-pFRS2α (1:1000), anti-pERK1/2 (1:1000), anti-ERK1/2 (1:1000), anti-pIKKα/β (1:1000), anti-pIKBα (1:1000), anti-IKBα (1:1000), anti-pp65 (1:1000), anti-p65 (1:1000), and anti-Vimentin (1:1000) were all purchased from Cell Signaling Technology (Danvers, MA); anti-COLA1 (1:1000), anti-ICAM1 (1:1000), anti-IL-1β (1:1000), anti-TIMP1 (1:1000), anti-TIMP2 (1:1000), and anti-TIMP3 (1:1000) were all obtained from Santa Cruz Biotechnology; and anti-GAPDH (1:500) was from Bioss (Beijing, CN). Specific bound antibodies were detected with horseradish peroxidase–conjugated goat anti-rabbit or anti-mouse IgG (1:10,000), and then visualized using the ECL detection kit. The images were analyzed using Image J software (NIH).

### SYTOX Green Staining

Cells plated in six-well tissue culture dishes were washed with distilled water. Subsequently, they were incubated for 20 min with 0.1 μM SYTOX Green (S7020, Thermo Fisher) to detect the DNA content in the treated cells. SYTOX green fluorescence was excited by 488 nm argon ion laser and the figure was captured by a fluorescence microscope (Nikon ECLIPSE TI-S).

### Gelatin Zymography

The activity of MMP-2 and MMP-9 was assessed by gelatin zymography. Briefly, the protein concentrations of tissue extracts were measured with the BCA protein assay kit (Thermo Fisher Scientific, Rockford, IL, United States). The samples containing 30 μg of proteins were separated on 10% PAGE containing 1 mg/ml gelatin (Sigma-Aldrich). After electrophoresis, the gels were treated with washing buffer containing 2.5% Triton X-100 (Sigma-Aldrich) for 1 h and then incubated for 24 h in a developing buffer [50 mM Tris, pH 7.6, 25 mM CaCl_2_, 0.2 mM NaCl, 0.02% (w/v) Brij-35] at 37°C. After the incubation, the gel was stained with Coomassie blue (Sigma Aldrich) and the gelatinolytic bands (MMP-2/MMP-9) were visualized in a dark blue background. MMP-2/MMP-9 in conditioned media of cells treated with TNFα and FGFRi were collected and analyzed by gelatin zymography as described above.

### ELISA Assay

Human IL-8 in HSC cultured medium and FGF21 in cellular lyses were measured by a competitive ELISA kit (IL-8 kit, Abcam, MA, United States; FGF21 kit, Biovendor, Modrice, Czech Republic). LX-2 were treated with TNFα and FGFR inhibitor in the wells of a six-well plate with 2 ml of DMEM. Twenty-four hours later, the conditioned medium was collected after centrifugation for the IL-8 ELISA analysis. The cell lysis was collected for FGF21 detection according to the manufacturer’s instructions.

### Gene Expression Analysis

Histological and ImmunohistochemicalTotal RNA was isolated from LX-2 cells and hepatic tissue using the Trizol RNA isolation reagent (Invitrogen, Carlsbad, CA). The SuperScript III reverse transcriptase (Invitrogen) was used for first-strand cDNA synthesis. Random primers were used for negative control according to the manufacturer’s instructions. Quantitative real-time polymerase chain reaction (PCR) was carried out using SYBR Green Master Mix with Roche LC480 light cycler system (Roche, Basel, Switzerland). The relative abundance of mRNA was calculated using the comparative threshold cycle method. β-Actin was used as an internal control for data normalization. Primers are listed in Table 1.

**TABLE 1:** Primer sequences for real time RT-PCR.

Primers	Forward	Reverse
h-GAPDH	ACAACTTTGGTATCGTGGAAGG	GCCATCACGCCACAGTTTC
h-FGF1	ACAGCCCTGACCGAGAAGTT	CCGTTGCTACAGTAGAGGAGT
h-FGF2	AGAAGAGCGACCCTCACATCA	CGGTTAGCACACACTCCTTTG
h-FGF7	TCCTGCCAACTTTGCTCTACA	CAGGGCTGGAACAGTTCACAT
h-FGF9	ATGGCTCCCTTAGGTGAAGTT	CCCAGGTGGTCACTTAACAAAAC
h-FGF10	CAGTAGAAATCGGAGTTGTTGCC	TGAGCCATAGAGTTTCCCCTTC
h-FGF21	GCCTTGAAGCCGGGAGTTATT	GTGGAGCGATCCATACAGGG
h-FGFR1	GGCTACAAGGTCCGTTATGCC	GATGCTGCCGTACTCATTCTC
h-FGFR2	GGTGGCTGAAAAACGGGAAG	AGATGGGACCACACTTTCCATA
h-FGFR3	CCCAAATGGGAGCTGTCTCG	CCCGGTCCTTGTCAATGCC
h-FGFR4	GAGGGGCCGCCTAGAGATT	CAGGACGATCATGGAGCCT
h-KLB	TTCTGGGGTATTGGGACTGGA	CCATTCGTGCTGCTGACATTTT
h-FRS2	CCTGCGACGCTATGGCTATG	ACGGGCACACTTAAAGGCAAA
h-MMP-1	AAAATTACACGCCAGATTTGCC	GGTGTGACATTACTCCAGAGTTG
h-MMP-2	CCCACTGCGGTTTTCTCGAA	CAAAGGGGTATCCATCGCCAT
h-MMP-3	CTGGACTCCGACACTCTGGA	CAGGAAAGGTTCTGAAGTGACC
h-MMP-9	AGACCTGGGCAGATTCCAAAC	CGGCAAGTCTTCCGAGTAGT
h-TIMP-1	CTTCTGCAATTCCGACCTCGT	ACGCTGGTATAAGGTGGTCTG
h-TIMP-2	GCTGCGAGTGCAAGATCAC	TGGTGCCCGTTGATGTTCTTC
h-TIMP-3	CAGGTCGCGTCTATGATGGC	AGGTGATACCGATAGTTCAGCC
h-IL-1β	TTCGACACATGGGATAACGAGG	TTTTTGCTGTGAGTCCCGGAG
h-IL-8	ACTGAGAGTGATTGAGAGTGGAC	AACCCTCTGCACCCAGTTTTC
h-ICAM-1	ATGCCCAGACATCTGTGTCC	GGGGTCTCTATGCCCAACAA
h-MCP-1	CAGCCAGATGCAATCAATGCC	TGGAATCCTGAACCCACTTCT
m-FGF21	CTGCTGGGGGTCTACCAAG	CTGCGCCTACCACTGTTCC
m-MMP-9	CTGGACAGCCAGACACTAAAG	CTCGCGGCAAGTCTTCAGAG
m-TNFα	CCCTCACACTCAGATCATCTTCT	GCTACGACGTGGGCTACAG
m-IL-6	CCAAGAGGTGAGTGCTTCCC	CTGTTGTTCAGACTCTCTCCCT

### Histological and Immunohistochemical Analyses

For MMP9 and p65 immunostaining, LX-2 cells were fixed with 4% paraformaldehyde for 2 h at room temperature. Antigens were retrieved by incubation in the 10 mM citrate buffer for 20 min. The cells were then incubated with rabbit anti-MMP-9 antibody (1:200, Abcam) or mouse anti-p65 (1:200, Cell Signaling Technology). The specifically bound primary antibodies were detected with Alexa 488-conjugated antirabbit or anti-mouse IgG antibodies (Thermo Fisher Scientific), respectively. The unbound antibodies were rinsed off by PBS. After counterstaining with DAPI for the nucleus, the images were captured with a confocal microscope (Zeiss LSM 510). For liver tissues, the sections were rehydrated and incubated with anti-neutrophil elastase (NE) (1:500) antibody overnight as described previously (Xin et al., 2007). Specifically bound antibodies visualized with the immunohistochemistry kit (MX Biotechnologies, Fuzhou, CN) according to the manufacturer’s protocol. Hematoxylin was used for counterstaining.

### Statistical Analyses

Survival time was analyzed on Kaplan–Meier survival plots using the log-rank test by SPSS software version 15.0 (SPSS Inc., IL, United States), Categorized variables were compared by chi square test (χ^2^) or Fisher’s exact test. *p* < 0.05 was considered to be statistically significant.

## Results

### Suppression of FGFR1 Kinase Compromises NF-κB Signaling Stimulated by TNFα in Human HSCs

**FIGURE 1:**
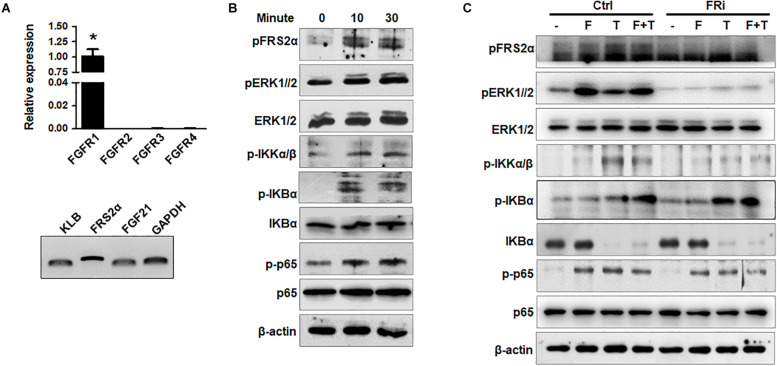
Disruption of FGFR1 signaling compromises NF-κB signaling stimulated by TNFα in human hepatic stellate cells. **(A)** RT-PCR analyses expression of FGFR isoforms and coreceptors in LX-2 cells. **(B)** LX-2 cells were treated with 50 ng/ml FGF1 for the indicated times. The cell lysates were analyzed by Western blot for activation of FGF and NF-κB signal pathways. **(C)** LX-2 cells were treated with the FGFR inhibitor (FRi), AZD4547, for 10 min and then 20 ng/ml FGF1 (F) and/or 10 ng/ml TNFα (T) for the indicated times. The cells were harvested for Western blot. β-Actin was used as an internal loading control. *p*FRS2, phosphorylated FRS2α; *p*ERK1/2, phosphorylated ERK1/2; *p*IKKα/β, phosphorylated IKKα/β; *p*IκBα, phosphorylated IκBα; *p*p65, phosphorylated p65. Data are expressed as mean ± SD; *P ≤ 0.05.

To investigate the crosstalk between the FGFR1 and NF-κB pathways in human HSCs, expression of FGFR isoforms in HSCs was firstly detected. Quantitative RT-PCR analyses revealed that LX-2 cells mainly expressed FGFR1. Expression of other FGFR isoforms was below the detection limit. FRS2α, an adaptor protein required for FGFR signal pathway activation, was highly expressed in LX-2 cells. The cells also expressed FGF21 and its coreceptor β-klotho (Figure 1A). Treating LX-2 cells with FGF1 induced strong phosphorylation of FRS2α and ERK1/2, as well as IKKα, IKKβ, IκBα, and p65, in LX-2 cells at a time-dependent manner (Figure 1B). These results indicate the association between the FGFR and NF-κB signal pathways, which is consistent with our previous report (Wang et al., 2018). Next, we investigated whether FGFR kinase activity was required for augmenting NF-κB signaling; LX-2 cells were treated with AZD4547 for 3 h before FGF treatment. The TNFα-induced activation of the NF-κB pathway was evaluated by Western blot analyses. The results showed that AZD4547 significantly decreased phosphorylation of IKKα/β and p65 and increased IκBα abundance (Figure 1C), suggesting that the activity of TNFα to activate NF-κB pathway was impaired.

### Inhibition of FGFR1 Reduces TNFα-Induced Apoptosis in LX-2 Cells

**FIGURE 2:**
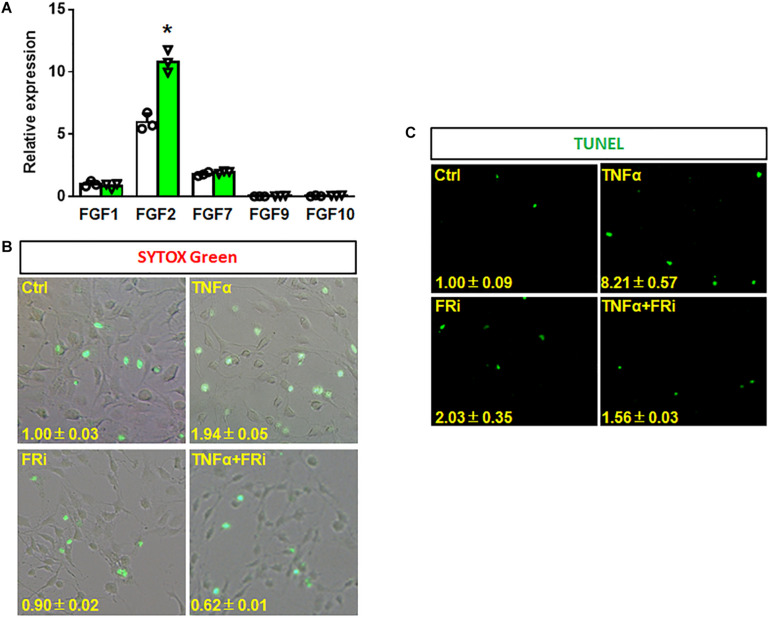
Inhibition of FGFR1 reduces TNFα-induced apoptosis in LX-2 cells. **(A)** RT-PCR analyses expression of FGFs in LX-2 cells after TNFα treatment. **(B, C)** LX-2 cells were treated with the FGFR inhibitor, AZD4547, for 10 min and then 20 ng/ml FGF1 and/or 10 ng/ml TNFα for 24 h. The cells were stained with SYTOX Green **(B)** and TUNEL **(C)** as the protocol. Data are expressed as mean ± SD; *P ≤ 0.05. Ctrl, solvent control; FRi, FGFR inhibitor, AZD4547.

To investigate the function of FGF in HSCs during liver injury, real-time RT-PCR was performed to determine expression of FGF related to HSCs. The results showed that FGF1, FGF2, and FGF7 were expressed in LX-2 cells. Interestingly, FGF2 expression was increased by one-fold after TNFα treatment (Figure 2A). To investigate whether FGFR1 interruption could affect cell viability after inflammatory stimulation, SYTOX Green staining and TUNEL were used to assess the cell vitality and apoptosis of LX-2. The results indicated that the number of apoptotic cells was significantly upregulated in the TNFα-induced group compared with the control group. The increases were diminished by treating with the FGFR inhibitor (Figure 2B). Consistent with SYTOX green staining results, TUNEL assay also showed that numbers of apoptotic cells were significantly reduced in the FGFR inhibitor-treated group compared with the TNFα-only group (Figure 2C).

### Inhibition of FGFR1 Suppresses the Activity of LX-2 Cell to Secrete Cytokines and Attract T Cells

**FIGURE 3:**
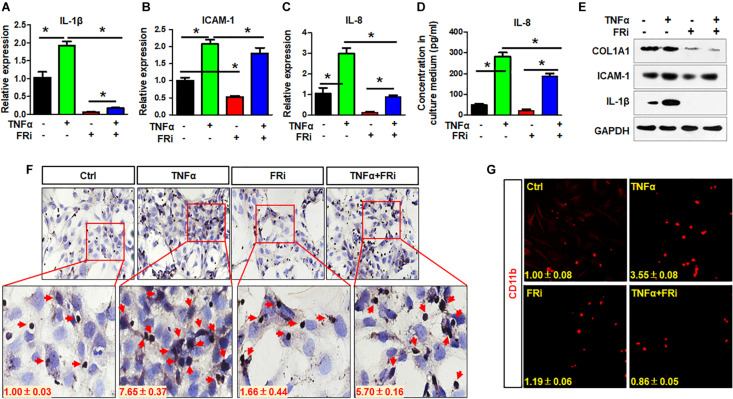
Inhibition of FGFR1 suppresses the activity of LX-2 cell to secrete cytokines and attract T cells. LX-2 cells were treated with the FGFR inhibitor, AZD4547, for 10 min and then 10 ng/ml TNFα for 24 h. Several proinflammatory cytokines IL-1β **(A)**, ICAM-1 **(B)**, and IL-8 **(C)** were examined by real-time PCR in LX-2 cells. **(D)** ELISA analysis confirmed the secreted IL-8. **(E)** The presence of indicated proteins was detected by Western blotting. **(F)** The trans-well assays were used to assess T cell migration in treated LX-2 cells with immunostaining by anti-CD3 antibody. Red arrows indicate T cells. **(G)** The cells were fixed and immunostained with anti-CD11b antibody. Red colors are CD11b-positive cells. Data are expressed as mean ± SD; *P ≤ 0.05. Ctrl, solvent control; FRi, FGFR inhibitor.

Inflammatory reaction is one of the earliest events in liver damages, which contributes to HSC activation. In return, activated HSCs also produce cytokines that promote inflammation. Therefore, expression of critical proinflammatory cytokines was assessed in LX-2 cells with or without treating with TNFα and FGFR inhibitor. Quantitative RT-PCR revealed that the expressions of key inflammatory cytokines, including IL-1β, ICAM-1, and IL-8, were induced by TNFα, and that the expression of the three cytokines were significantly reduced by treating with FGFR inhibitor (Figures 3A–C). Consistently, ELISA analyses demonstrated that secretion of IL-8 to the medium was reduced by the FGFR inhibitor (Figure 3D). Western blot also showed that TNFα-induced IL-1β and ICAM-1 expression was significantly decreased in the group treated with FGFR inhibitor (Figure 3E). The results suggest that inhibition of FGFR signaling represses inflammation after acute hepatic injury through suppressing release of proinflammatory cytokines by HSCs.

To determine whether suppression of FGF signaling compromised the activity of LX-2 cells to recruit T cells, the trans-well assays were used to assess LX-2-induced T cell migration with and without treating with TNFα and FGFR inhibitor (Figure 3F). It is clear that TNFα promoted LX-2 to recruit T cells. However, the activity was blunted by treating with FGFR inhibitors (Figure 3F). Similar results were observed with monocyte recruitment (Figure 3G). These results further demonstrate the crosstalk between FGF and NF-κB pathways in regulating inflammatory response.

### Inhibition of FGFR1 Compromises Expression and Activation of MMP-9 in Activated HSCs

**FIGURE 4:**
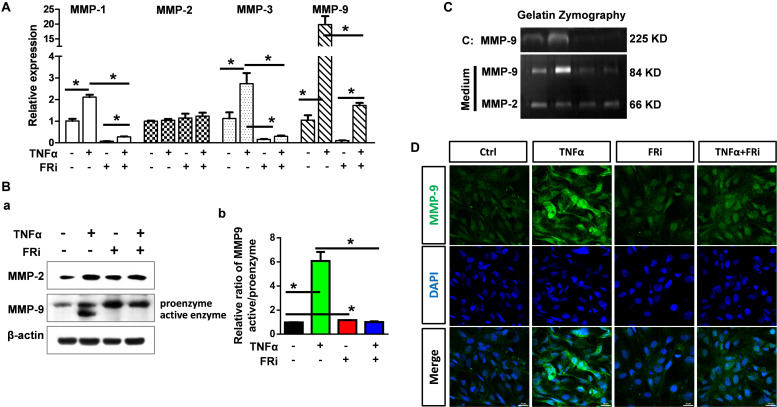
Inhibition of FGFR1 compromises expression and activation of MMP-9 in activated HSCs. LX-2 cells were treated with the FGFR inhibitor, AZD4547, for 10 min and then 10 ng/ml TNFα for 24 h. **(A)** RNA was extracted for RT-PCR analyses to determine the expression of MMP-1, MMP-2, MMP-3, and MMP-9. **(B)** The cells were lysed and analyzed by Western blotting to determine the protein levels of MMP-2 and MMP-9 (a), the relative ratio of activated-MMP-9 compared with proenzyme (b). **(C)** Gelatin zymography analyzed the activation of MMP-2 and MMP-9. **(D)** The cells were fixed and immunostained with anti-MMP-9 antibody. Data are normalized with a β-actin control and expressed as mean ± SD; *P ≤ 0.05. Ctrl, solvent control; FRi, FGFR inhibitor, AZD4547.

To determine how TNFα affected the function of MMPs in acute hepatitis, we assessed the expression and activation of MMPs and the broad MMP inhibitor, TIMPs. Quantitative RT-PCR showed that TNFα significantly increased MMP9 expression in LX-2 cells. The induction of MMP1 and MMP3 expression was moderate. No difference was observed in MMP2 expression. Treating the cells with FGFR inhibitors largely blunted the increase (Figure 4A). Western blot confirmed increased MMP9 expression at the protein level. Furthermore, both Western and zymography analyses revealed that TNFα induced activation of MMP9 in LX-2 cells. Treating of the cells with FGFR inhibitor abrogated the activation (Figures 4B,C). Immunostaining also showed that TNFα-induced MMP9 expression was suppressed by FGFR inhibitors (Figure 4D).

**FIGURE 5:**
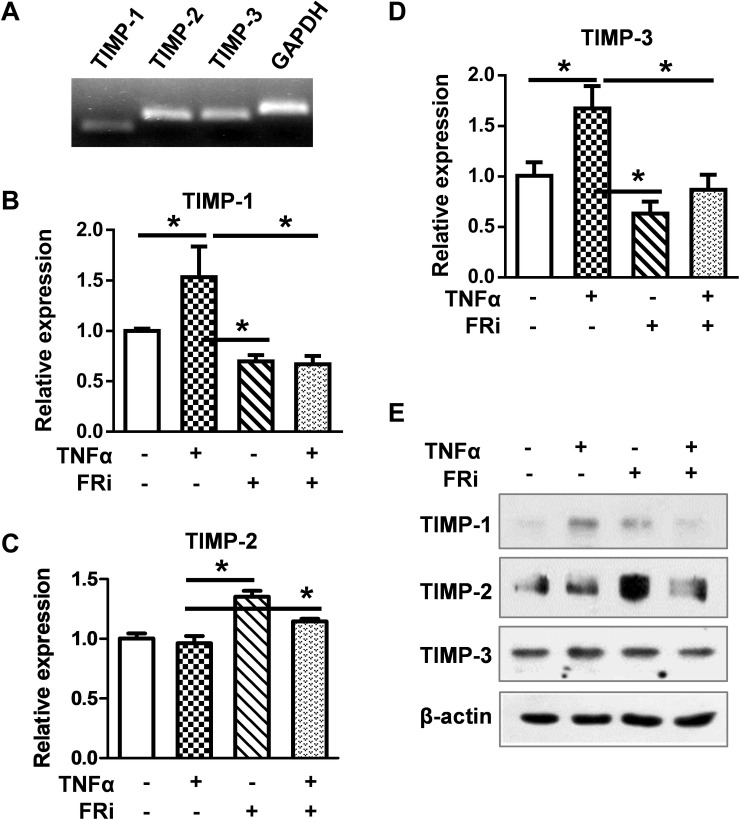
Inhibition of FGFR1 reduces TIMP expression in LX-2 cells. LX-2 cells were treated with the FGFR inhibitor, AZD4547, for 10 min and then 10 ng/ml TNFα for 24 h. The cells were harvested for **(A)** RT-PCR analysis, which detects the endogenous expression of TIMPs by agarose gel electrophoresis. **(B–D)** Real-time RT-PCR was used to analyze the mRNA levels of TIMP1/2/3 in indicated groups. **(E)** Western blotting analyses were taken to confirm the protein expressions of TIMPs. Data are normalized with a β-actin control and expressed as mean ± SD; *P ≤ 0.05. Ctrl, solvent control; FRi, FGFR inhibitor, AZD4547.

To determine whether FGF signaling also regulated TIMPs expression, real-time RT-PCR and Western blot were used to detect the expression of TIMP1, TIMP2, and TIMP3. The results showed that TNFα induced TIMP-1 expression at the mRNA and protein levels. Treating the cells with FGFR1 inhibitor diminished the increases (Figure 5), indicating that FGFR1 signaling is required for TNFα to promote TIMP1 expression. However, the expression of TIMP2 and TIMP3 was not consistent, implying that FGFR1 signaling was not involved in TIMP2 and TIMP3 expression in LX-2 cells. Together, these results reveal that the expression and activation of MMP are regulated by the crosstalk between FGF and TNFα pathways in LX-2 cells.

### Suppression of FGF Signaling Dampens Activation of NF-κB

**FIGURE 6:**
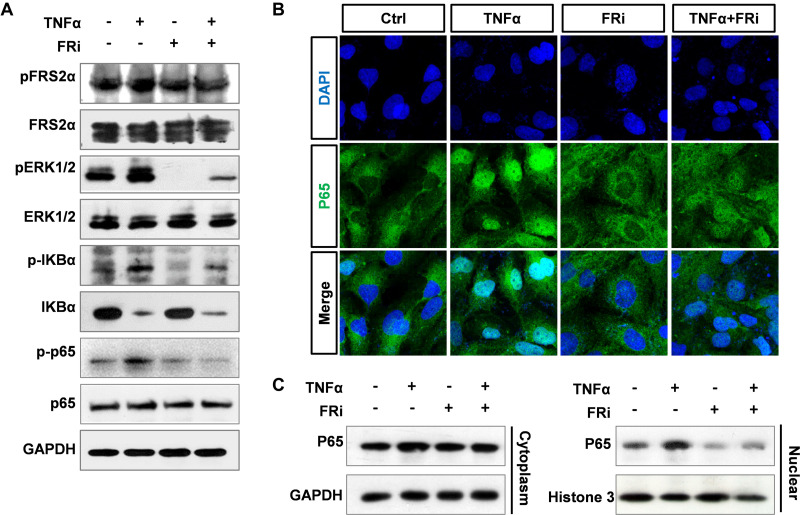
Suppression of FGF signaling dampens activation of NF-κB in LX-2 cells. LX-2 cells were treated with the FGFR inhibitor, AZD4547, for 10 min and then 10 ng/ml TNFα for 24 h. **(A)** The cells were harvested for Western blot. GAPDH was used as an internal loading control. *p*FRS2, phosphorylated FRS2α; *p*ERK1/2, phosphorylated ERK1/2; *p*IκBα, phosphorylated IκBα; *p*p65, phosphorylated p65. **(B)** The cells were fixed and immunostained with anti-p65 antibody to detect p65 cellular location. **(C)** The cells were lysed, and the lysates were separated into cytosol and nuclear fractions. The abundance of p65 in the two fractions was assessed by Western blotting. Data are normalized with a GAPDH and/or Histone 3 control. Ctrl, solvent control; FRi, FGFR inhibitor, AZD4547.

It has been documented that TNFα induces MMP9 expression in multiple cell types, via promoting the binding of P65 to the MMP9 promoter (Liu et al., 2016). To determine whether FGF promoted MMP9 expression via the NF-κB pathway, LX-2 cells were treated with TNFα with or without cotreatment with FGFR inhibitors. Western blotting showed that the increased expressions of phospho-FRS2α, phospho-ERK1/2, phospho-IκBα, and phospho-p65 were markedly decreased compared with TNFα treatment alone. As degradation of IκBα is associated with NF-κB activation and conversely the increase is correlated with NF-κB inhibition, we found that the increased protein level of IκBα in HSCs treated with FGFRi was consistent with NF-κB suppression (Figure 6A). In addition, both immunostaining and Western blot revealed that TNFα-induced nuclear translocation of p65 was reduced in LX-2 cells (Figures 6B,C). The results indicate that inhibition of FGF signaling reduces MMP9 expression, at least in part, via dampening the NF-κB activation.

### FGF21 Attenuates Con A-Induced Acute Liver Inflammation in Mice

**FIGURE 7:**
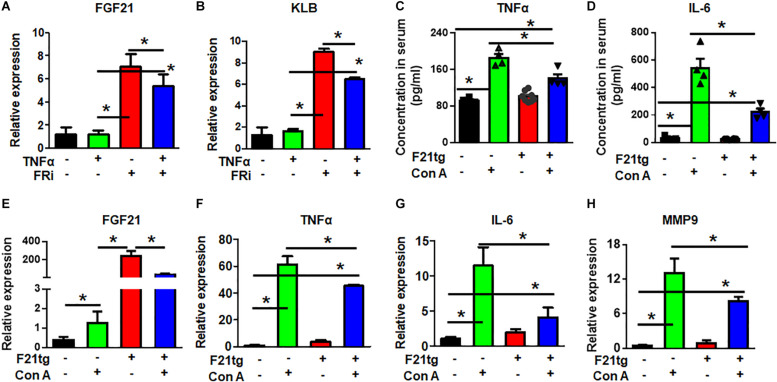
FGF21 attenuates of Con A-induced acute liver inflammation. LX-2 cells were treated with the FGFR inhibitor, AZD4547, for 10 min and then 10 ng/ml TNFα for 24 h. RT-PCR analyses expression of FGF21 and KLB **(A,B)**. Wild-type and FGF21 transgenic mice were injected with saline or Con A via the tail vein for 8 h, and the levels of TNFα **(C)** and IL-6 **(D)** in serum were detected by ELISA analysis. The liver was harvested for quantitative RT-PCR analyses for FGF21 **(E)**, TNFα **(F)**, IL-6 **(G)**, and MMP-9 **(H)** expression at the mRNA level. Data are expressed as mean ± SD; *P ≤ 0.05. FRi, FGFR inhibitor, AZD4547; F21tg, FGF21 transgenic mice.

Real-time RT-PCR showed that LX-2 cells expressed endogenous FGF21. Surprisingly, the expression of FGF21 and its co-receptor, β-klotho (KLB), was significantly increased by treating with FGFR inhibitors, suggesting a negative feedback control by FGF signaling. However, the increased FGF21 and KLB expression was reduced in the TNFα and FGFR inhibitor-treated group (Figures 7A,B). The results further demonstrate the crosstalk between the two pathways.

To investigate how FGF21 regulated the inflammation in the hepatitis milieus, wild-type and transgenic mice expressing FGF21 in the liver were treated with Con A to induce acute live injury via tail vein injection. The liver was dissected 8 h after the injection. As expected, Con A treatment increased serum TNFα and IL-6 concentrations. However, the folds of increase were significantly reduced in FGF21 transgenic mice (Figures 7C,D). Interestingly, the expression of endogenous FGF21 was upregulated in the Con A-treated liver (Figure 7E), suggesting that increase of FGF21 is a stress response. Consistently, Con A injection increased TNFα, IL-6, and MMP9 expression in the liver, and the increase was diminished in FGF21 transgenic mice (Figures 7F–H).

**FIGURE 8:**
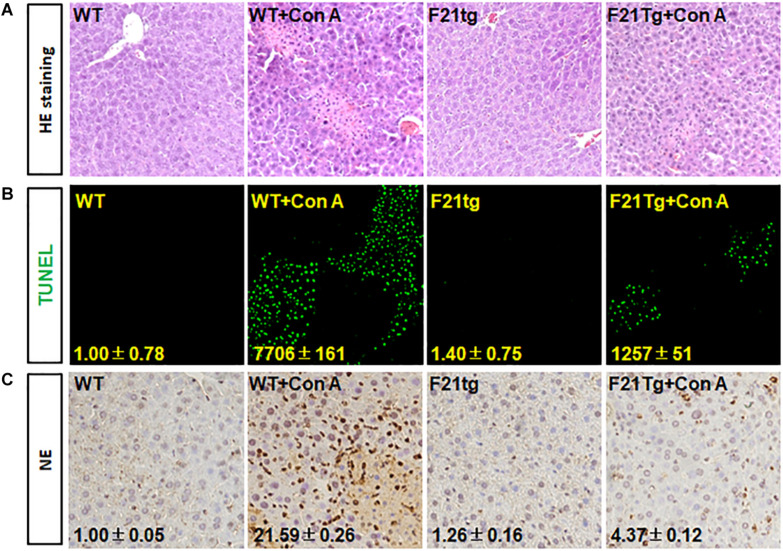
FGF21 overexpression reduces apoptosis in Con A-injured liver. Wild-type and FGF21 transgenic mice were injected with saline or Con A via the tail vein for 8 h. **(A)** H&E staining of liver tissue sections derived from mouse model with or without Con A induced. **(B)** TUNEL assay was used to detect the apoptosis in liver tissues. **(C)** Tissue sections were immunostained with anti-NE antibody to check the neutrophil infiltration in Con A-induced liver microenvironment. WT, wild-type mice; F21tg, FGF21 transgenic mice.

In addition, H&E staining revealed that compared with wild-type mice, the liver injury in FGF21 transgenic mice was less severe (Figure 8A). Consistently, TUNEL analyses showed that the transgenic liver had less apoptotic cells (Figure 8B). Moreover, immunostaining with the anti-NE antibody showed that FGF21 transgenic liver had less neutrophil infiltration than that of control mice (Figure 8C). Together, these results demonstrate that overexpression of FGF21 in the liver decreases liver damage induced by Con A.

## Discussion

**FIGURE 9:**
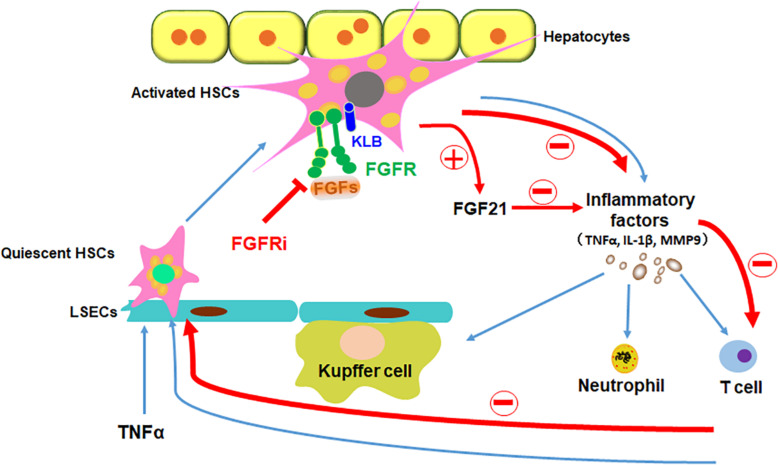
Schematic diagram of FGFR1 inhibition alleviates inflammatory responses in the injured liver. TNFα induces inflammation in liver via stimulating quiescent HSCs in the sinusoids to be transdifferentiated to activated HSCs, which secrete inflammatory factors to recruit leukocytes to the liver. Disruption of FGF signaling with FGFR inhibitor could ameliorate inflammatory response in HSCs in the ways of decreasing proinflammatory cytokine secretion (such as TNFα, IL-1β, and MMP9), reducing immune cells (such as Kupffer cells, neutrophils, and T cells) recruitment and inhibiting NF-κB translocation in the liver; in addition, increase of FGF21 secretion from HSCs directly restrains the inflammatory reaction. In conclusion, suppressing canonical FGF signaling downregulates sinusoidal inflammation in the liver, which is presented in all stages of diseased liver.

In addition to being an organ for metabolism and detoxification, the liver is also an organ where immune responses take place. In the liver, sinusoids directly connected to the portal circulation serve as the first barrier against noxious stimuli. It contains sinusoidal endothelial cells, Kupffer cells, HSCs, and others (Bottcher et al., 2018). Treating HSCs with proinflammatory cytokines and LPS results in the activation of proinflammatory signaling pathways, including the NF-κB pathway, and subsequent production of chemokines and cytokines (Chen et al., 2017; Kim et al., 2019). It has been predicted that HSCs regulate inflammation during acute liver injury (Breitkopf-Heinlein et al., 2017). In the present study, we showed that inhibition of FGFR compromised TNFα-induced activation of the NF-κB pathway in HSCs and suppresses the inflammation induced by acute liver injuries. Interestingly, activation of HSCs increased endogenous FGF21 expression. Overexpression of FGF21 reduced the magnitude of inflammation, together with a previous report that FGFR elicits a different set of signals with and without the coreceptor, KLB. Therefore, the results suggest that FGFR1–heparan sulfate complex activated by FGF2 may release non-canonical signaling that promotes inflammation as we reported earlier (Wang et al., 2018), the FGFR1–KLB complex activated by FGF21 may elicit anti-inflammatory signals. Thus, the data further demonstrate that the FGFR and FGFR–KLB elicits a distinctive set of signals (Figure 9).

Our data showed that FGF2 expression is upregulated two-fold compared with control by TNFα. Unlike FGF21 that only activates the FGFR–KLB complex and elicits anti-inflammation, FGF2 activates the FGFR–heparan sulfate complex and stimulates inflammation. Therefore, it is important to selectively suppress FGF2 activated pathways, rather than simply inhibiting the tyrosine kinase activity, to reduce liver damage after acute liver injury. Further efforts are warranted to identify the specific pathway(s) of FGFR1 that promotes the inflammation. Equally important, it is urgently needed to identify the pathway(s) used by the FGFR–KLB receptor complex that is activated by FGF21.

During liver injury, HSCs are activated by molecules released from the surrounding cells, including hepatocytes, Kupffer cells, endothelial cells, leukocytes, and platelets (Stewart et al., 2014). These molecules include cytokines, lipid peroxides, growth factors, and reactive oxygen species, which regulate HSC activities and proliferation (Tsuchida and Friedman, 2017). In addition, bone marrow-derived monocytes massively accumulate in the injured liver and differentiate into inflammatory macrophages (Milosevic et al., 2019). Neutrophils are the rapid and first line of defense against many pathogenic microorganisms. They express multiple molecules that regulate inflammatory and immune responses (Jaeschke et al., 1996; Gehrke et al., 2018). Our work revealed that neutrophils were recruited to the liver after the Con A-induced hepatic injury. These infiltrated inflammatory cells robustly secreted proinflammatory cytokines, including IL-1β, ICAM-1, and IL-8, and induced MMP9 expression in the injured liver. Although the mechanisms are not fully understood, our data showed that suppression of FGFR restrained the inflammation and therefore reduced the damaged in the injured liver via dampening the TNFα–NF-κB pathways.

In a TNFα-induced acute hepatitis mouse model, the lethality, hypothermia, and influx of leukocytes into the liver are all suppressed by MMP inhibitors, indicating that MMPs play important roles in inflammation associated with acute hepatitis (Nicoud et al., 2007). In our report, we showed that, in TNFα-stimulated HSCs, MMP-1, MMP-2, MMP-3, and MMP-9 were increased at the transcriptional level after TNFα induction and that suppression of FGFR kinase diminished the increases. Among the MMPs, MMP-9 was the most abundant isoform in active HSCs. In addition, although all MMP cognate inhibitors are expressed in LX-2 cells, only TIMP-2 expression at the protein levels are negatively regulated by FGFR. This suggests that MMP9 plays an important role in the crosstalk between TNFα and FGF pathways during acute liver injury.

Con A damages the sinusoidal endothelial cell barrier, leading to the direct exposure of HSC and hepatocytes to cytokines and sinusoidal cells (Gong et al., 2015). Inflammatory cytokines then recruit inflammatory cells to the liver and cause massive liver injury (Chen et al., 2016). FGF21 has been reported to regulate HSC activation, apoptosis, and development of fibrosis in both gain-of-function and loss-of-function studies. In the gain-of-function study, FGF21 suppresses the development of hepatic inflammation and the expression of inflammatory molecules, including the NF-κB pathway, at the protein and mRNA levels (Kang et al., 2018). Subcutaneous infusion of FGF21 reverses the increased expression of profibrotic and proinflammatory genes in FGF21 deficient mice (Geller et al., 2019). Using the transgenic mouse model that overexpresses FGF21 in the liver, we demonstrated that overexpression of FGF21 blunted Con A-induced leukocyte recruitment to the liver and reduced the liver injury and hepatocyte apoptosis. There results demonstrated that FGF21 is a protective factor in Con A-induced hepatitis, although the detailed mechanism needs to be further investigated in the future.

In this report, we demonstrated that suppression of FGFR in activated HSCs compromises the activation of NF-κB by TNFα and MMP9 expression and activation. The results demonstrate the proinflammation role of FGFR kinase. We also showed that overexpression of FGF21 reduced inflammation and infiltration of immune cells into the liver upon Con A-induced hepatitis. As FGFR elicits a different set of signals upon complexing with KLB, it is important to unravel the detailed pathways that FGFR uses to promote inflammation and FGFR–KLB uses to suppress inflammation during acute liver injury. The results also reveal a therapeutic value of FGF21 in treating acute liver injury.

## Data Availability Statement

All datasets generated for this study are included in the article/supplementary material.

## Ethics Statement

The animal study was reviewed and approved by the Program of Animal Resources of the Wenzhou Medical University.

## Author Contributions

CW and XP: conceptualization, resources, and writing – review and editing. YL, HL, ZY, YZ, XW, and ZF: methodology and validation. YL, HL, and ZY: software. YL, HL, and ZF: formal analysis. CW and XL: investigation. ZF, TZ, WZ, and XW: data curation. YL, XP, and CW: writing – original draft. CW and JM: project administration. JM, CW, and XL: funding acquisition. All authors contributed to the article and approved the submitted version.

## Conflict of Interest

The authors declare that the research was conducted in the absence of any commercial or financial relationships that could be construed as a potential conflict of interest.
